# XROMM and diceCT reveal a hydraulic mechanism of tongue base retraction in swallowing

**DOI:** 10.1038/s41598-020-64935-z

**Published:** 2020-05-19

**Authors:** Courtney P. Orsbon, Nicholas J. Gidmark, Tingran Gao, Callum F. Ross

**Affiliations:** 1 Department of Organismal Biology & Anatomy, The University of Chicago, Chicago, IL 60637 USA; 2 Biology Department, Knox College, Galesburg, IL 61401 USA; 3 Committee on Computational and Applied Mathematics, Department of Statistics, The University of Chicago, Chicago, IL 60637 USA

**Keywords:** Experimental organisms, Imaging, Feeding behaviour, Bone quality and biomechanics, Biological anthropology, Biological techniques, Physiology, Experimental models of disease, Preclinical research

## Abstract

During primate swallowing, tongue base retraction (TBR) drives the food bolus across the oropharynx towards the esophagus and flips the epiglottis over the laryngeal inlet, protecting against penetration and aspiration of food into the airway. Despite the importance of TBR for swallowing performance, the mechanics of TBR are poorly understood. Using biplanar videoradiography (XROMM) of four macaque monkeys, we tested the *extrinsic muscle shortening hypothesis*, which posits that shortening of the hyoglossus and styloglossus muscles pulls the tongue base posteriorly, and the *muscular hydrostat* or *intrinsic tongue muscle hypothesis*, which suggests that, because the tongue is composed of incompressible fluid, intrinsic muscle shortening increases tongue length and displaces the tongue base posteriorly. Our data falsify these hypotheses. Instead we suggest a novel *hydraulic mechanism* of TBR: shortening and rotation of suprahyoid muscles compresses the tongue between the hard palate, hyoid and mouth floor, squeezing the midline tongue base and food bolus back into the oropharynx. Our hydraulic mechanism is consistent with available data on human tongue swallowing kinematics. Rehabilitation for poor tongue base retraction might benefit from including suprahyoid muscle exercises during treatment.

## Introduction

Retraction of the tongue base against the posterior pharyngeal wall is vital a part of swallowing in mammals, including humans^1–4^ but how this retraction happens is not well understood. Two mechanisms have been hypothesized. The ***extrinsic muscle shortening hypothesis*** posits that contraction of the hyoglossus and styloglossus muscles pulls the tongue base posteriorly^4–9^. In support of this hypothesis, the lines of action of the styloglossus and hyoglossus muscles both have posteriorly-oriented components and these muscles are active during swallowing in many mammals, including humans^9–13^. The ***muscular hydrostat*** **or** ***intrinsic tongue muscle hypothesis*** suggests that, because the tongue is largely composed of incompressible fluid, reduction in tongue base width due to contraction of the transversely oriented intrinsic tongue muscles must be associated with increases in posterior tongue length, and hence tongue base retraction (TBR)^6,14,15^. In support of this mechanism, dynamic magnetic resonance imaging (MRI) of humans during swallowing reveals increases in posterior tongue length and depth that are hypothesized to be caused by decreases in tongue width^6–8^.

Despite widespread acceptance of these two hypotheses, both mechanisms rest on untested assumptions. Contraction (shortening) of styloglossus, palatoglossus, and hyoglossus muscles during swallowing, which is central to the *extrinsic muscle shortening hypothesis*, has not yet been demonstrated. Although these muscles are active during swallowing^9,10,16^, muscle activity need not be associated with muscle shortening: claims of muscle function require measurements of both muscle activity and muscle velocity^17–20^. Moreover, styloglossus, palatoglossus and hyoglossus muscles insert into the lateral tongue, so if their retracting forces are to cause midline TBR, that force must be transmitted to the midline tongue base. However, the anatomy of human and macaque tongues suggests the presence of internal shearing planes between the lateral insertions of extrinsic tongue muscles to the back of the tongue and intrinsic muscles around the midline. This would effectively allow movement of the tongue midline to be at least partially independent of its lateral borders. Such independent movement would facilitate tongue bending in coronal and sagittal planes, as well as in the transverse planes already described^14^.

Similarly, mediolateral contraction of the intrinsic tongue base muscles, assumed by the *intrinsic muscle hypothesis* to be powering TBR, has yet to be demonstrated. Mediolateral contraction of intrinsic tongue base muscles will only displace the tongue base backwards in primates and humans if tongue volume is regionally conserved during TBR; i.e., the tongue functions as a muscular hydrostat. This seems to not be the case in pig tongues, which exhibit regional changes in volume during feeding^21,22^, raising doubts about the validity of the muscular hydrostat model.

**Figure 1:**
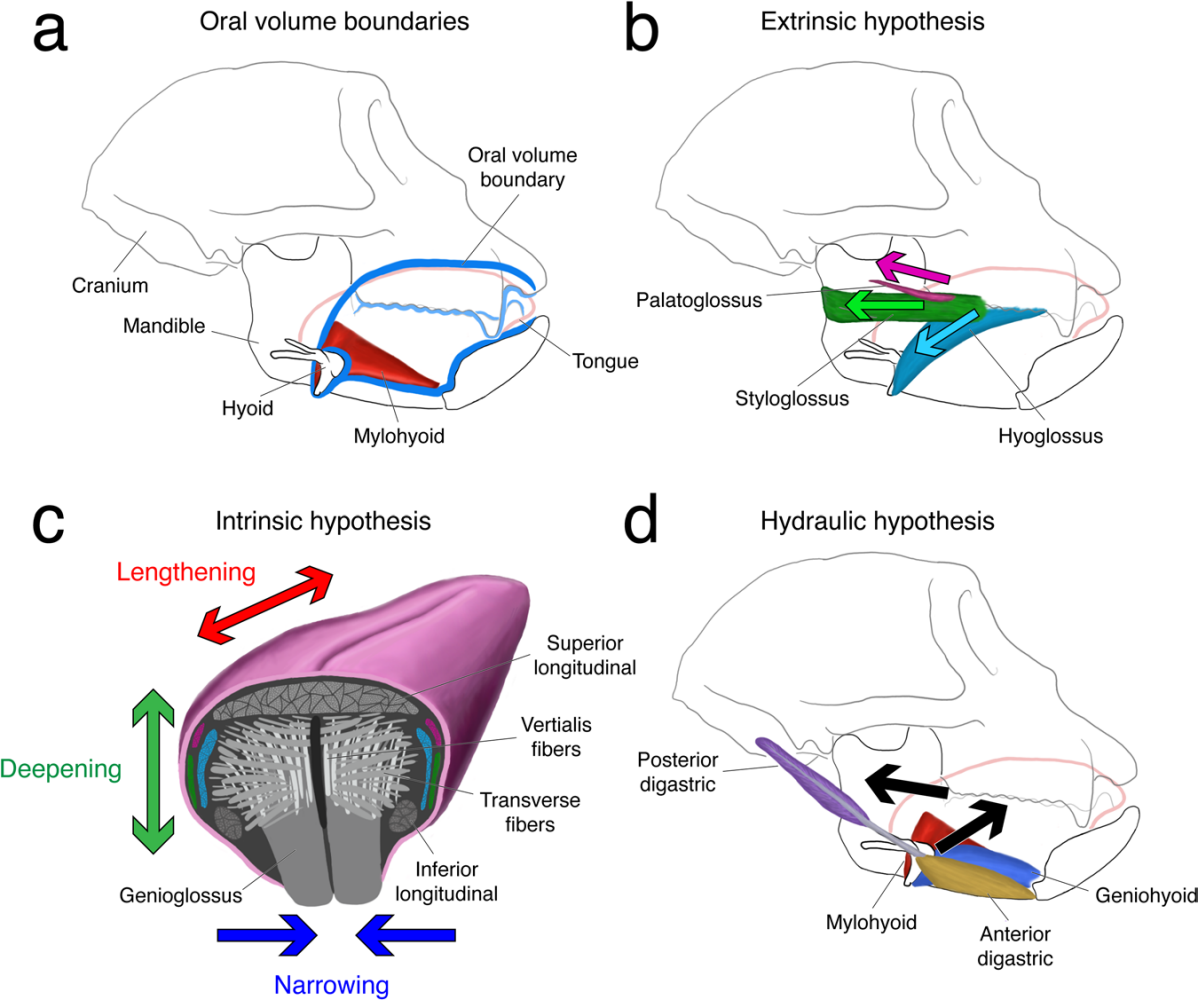
Hypothetical mechanisms of tongue base retraction (TBR) in isolation. (**a**) Anatomy of the macaque cranium, mandible, hyoid, and tongue (pink). The oral volume (blue) is a chamber composed of rigid hard palate, hyoid, mandible, and tooth rows, and floored by variably stiff suprahyoid muscles such as mylohyoid (red). The oral tongue is enclosed within this chamber when the jaws are closed, whereas the tongue base is free to expand posteriorly. Each of the following mechanisms is shown in isolation; however, these mechanisms are not mutually exclusive and tongue base retraction could occur through a combination of the three. (**b)** Illustration of the extrinsic muscle shortening hypothesis. The hyoglossus (light blue), palatoglossus (dark pink), and styloglossus (green) muscles all have posteriorly-oriented trajectories throughout swallowing and could each theoretically cause TBR. (**c)** Illustration of the intrinsic muscle shortening hypothesis, showing a primate tongue in coronal cross-section. Colors for extrinsic tongue muscles correspond to those in b. Assuming that the tongue behaves as a classic muscular hydrostat, intrinsic tongue muscles (shades of gray) – specifically, the transverse fibers – shorten to produce narrowing (blue). If regional volume is conserved, then this narrowing produces simultaneous lengthening (red) and deepening (green) of the tongue. (**d)** Illustration of the hydraulic hypothesis. Hyoid protraction by geniohyoid (blue) and elevation of the mouth floor by co-contraction of mylohyoid (red) and the digastric bellies (anterior, yellow; posterior, purple) produce a net anterosuperior movement of the hyoid and mouth floor that encroach into the oral volume containing the tongue. When the tongue is incompressible within the confines of the oral volume, its base must displace posteriorly–TBR.

Hydraulic designs utilize “displacement of fluid from one location in order to cause shape change or exert force elsewhere”^23^. For shape change or force to be generated in one part of a hydraulic structure, they need to be resisted elsewhere, often through connective tissues with various degrees of rigidity in various orientations. Hydraulic structures are common among invertebrates and vertebrates, such as echinoderm tube feet and tumescent vertebrate penises, in which connective tissue sheets provide resistance and  muscular contraction or increased bloodflow provides the driving force. In human and non-human primates (macaques), the oral volume housing the tongue (Fig. 1a) is a constricted chamber composed of rigid hard palate, hyoid, mandible, and tooth rows. When the lower jaw is elevated the oral volume is floored by suprahyoid muscles–digastrics,

mylohyoid, geniohyoid, hyoglossus, stylohyoid muscles—which, depending on their levels of activity, can variably stiffen and shorten, to modulate pressure in the oral volume. These structures have also been hypothesized to drive specialized aspects of tongue function in at least one species of mammal^23–27^ but their influence on tongue deformation, particularly tongue base retraction mechanics, is largely unstudied.

This uncertainty led us to develop a third hypothesis*—*the ***hydraulic hypothesis****—*which posits that hyoid protraction and mouth floor elevation cause TBR via a hydraulic linkage between the tongue and suprahyoid muscles. In this hypothesis, midline tongue elevation and retraction can occur independent of active intrinsic or extrinsic tongue muscle shortening because it is contraction of suprahyoid muscles that drives TBR. Such a mechanism would couple suprahyoid muscle shortening and consequent hyoid excursion to TBR during the oral phase of swallowing: the suprahyoid muscles stiffen and elevate the mouth floor, causing the hyoid and mouth floor to encroach on the oral volume, therefore decreasing space available for the tongue’s total volume and forcing the tongue base to retract via posterior volume displacement. In essence, we hypothesized that the hyoid apparatus functions as a piston for the tongue base, which itself functions as a piston for the bolus^1^.

To test these hypotheses, we combined *in vivo* bi-planar x-ray imaging with the XROMM workflow^28^ and contrast-enhanced high-resolution anatomical imaging (diceCT^29^) to quantify 3D kinematics of the jaw, hyoid, cranium, and tongue in macaque monkeys. Combining these imaging methodologies allowed us to measure length change in both intrinsic and extrinsic tongue muscles, six-degree-of-freedom movements of skeletal elements, and *regional* variation in tongue volume during swallowing at an unprecedented spatio-temporal scale^20^. We measured changes in lengths of the extrinsic muscles during swallowing to test the *extrinsic muscle shortening hypothesis* (Fig. 1b). This hypothesis is falsified if the tongue base retracts more than these muscles shorten, or (in the case of the hyoglossus muscle) if their lingual insertions do not retract as the muscles shorten. We also measured 3D changes in tongue shape (in three different segments) during swallowing to test the *intrinsic muscle shortening hypothesis* (Fig. 1c). This hypothesis is falsified if all three dimensions of the tongue base increase, and/or the tongue base increases in volume. Lastly, we compared the relative timing of changes in the regional volume of the tongue and oral cavity to test our *hydraulic hypothesis* (Fig. 1d). Although the method does not capture absolute tongue volume, if the tongue functions as a muscular hydrostat then the regional volume capture by our methods should remain constant or at least minimally variable. This hypothesis is falsified if regional tongue base volume does not increase as the hyoid elevates and protracts, the mouth floor elevates, and the oral volume decreases.

## Results

A total of 99 swallow cycles was analyzed including 85 intercalated swallows—between chewing cycles—and 14 terminal swallows—at the end of a feeding sequence. Pooled standard deviations for all measured variables across all four animals, determined by a frozen specimen precision experiment^30^, are documented in Table 1. Terminal swallows were broadly similar to intercalated swallows, save for less variation and increased tongue base retraction in terminal swallows (see Supplement).

**Table 1:** Precision* across all four animals in a cranial coordinate system.

Markers (X | Y | Z, mm)		Muscle Length (mm)		Tongue Dimensions (mm)		Volume (mL)	
Anterior	0.07 | 0.08 | 0.07	Hyoglossus	0.06	Anterior Depth	0.04	Anterior Oral Tongue	0.006
Lateral Right	0.07 | 0.05 | 0.07	Palatoglossus	0.04	Anterior Length	0.05	Posterior Oral Tongue	0.021
Lateral Left	0.07 | 0.05 | 0.06	Styloglossus	0.05	Anterior Width	0.06	Tongue Base	0.005
Middle Surface	0.06 | 0.05 | 0.06	**Muscle Orientation (°)**		Posterior Depth	0.05	Oral Volume	0.077
Middle Deep	0.07 | 0.05 | 0.06	Hyoglossus	0.12	Posterior Length	0.05	**Mandible Kinematics (°)**	
Posterior Right	0.06 | 0.04 | 0.06	Palatoglossus	0.27	Posterior Width	0.07	Pitch	1.61
Posterior Left	0.06 | 0.05 | 0.06	Styloglossus	0.10	Tongue Base Depth	0.05		
Posterior Surface	0.06 | 0.03 | 0.05			Tongue Base Length	0.04	**Hyoid Kinematics (mm)**	
Posterior Deep	0.07 | 0.04 | 0.05			Tongue Base Width	0.05	Protraction	0.10
Vallecula	0.06 | 0.05 | 0.04					Elevation	0.04

*As measured by the pooled standard deviation, ${s}_{pooled}=\sqrt{\frac{{s}_{1}^{2}+{s}_{2}^{2}+\mathrm{..}.+{s}_{k}^{2}}{k}}$ where *s* is the standard deviation and k is the number of animals.

### The majority of tongue movement is in the posterior midline and concurrent with hyoid excursion

**Figure 2:**
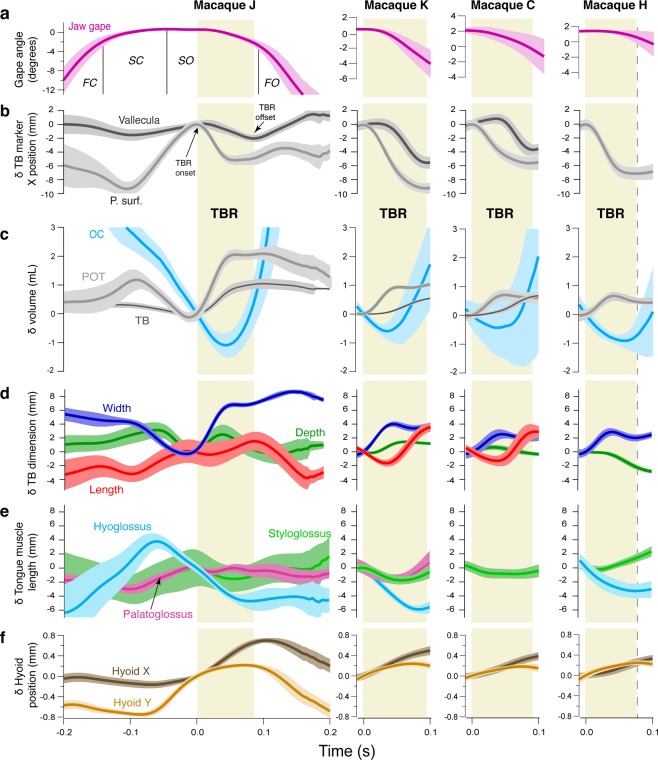
Jaw and hyolingual kinematics during tongue base retraction (TBR) phase of swallow gape cycles. Traces are mean values +/− 1 SD. Gape angles are unadjusted means; all other variables are changes (δ) relative to the value at start of TBR. TBR was defined as the time during the swallow cycle starting when the posterior superficial tongue marker *started* moving posteriorly relative to the cranium until the time when the vallecular marker *stopped* moving posteriorly. All data are in the coordinate system of the cranium. (**a)** Jaw gape angle (in degrees). Y axis for animal J covers the full range of jaw gape angles. Y axes for animals K, C, H maximize resolution during TBR. A pitch angle of 0° or less indicates that the mandibular cusps are superior to the cranial cusps, i.e., intercuspation. (**b)** Change (δ) in X position (anteroposterior) of posterior superficial (P. surf.) and vallecular tongue markers in the tongue base. (**c)** Change (δ) in volume of oral cavity (OC), posterior oral tongue volume (POT), and tongue base volume (TB). Oral volume was modeled using an alpha hull with alpha = 6; tongue volume was modeled using meshes. (**d)** Change (δ) in TB dimensions, width, length, depth (mm). E. Change (δ) in tongue extrinsic tongue muscle lengths: styloglossus, hyoglossus, palatoglossus (mm). F. Change (δ) in hyoid X (anteroposterior) and Y (superoinferior) position. Data for Monkey J are given for the whole swallow cycle. Data for Monkeys K, C and H are given for the period from TBR onset (0.0 s) to 0.1 s. TBR phase is indicated by the pale yellow rectangles. Dashed line at offset of TBR in Monkey H indicates no vallecular marker data were available. TBR offset estimated as end of posterior superficial marker posterior movement.

**Figure 3:**
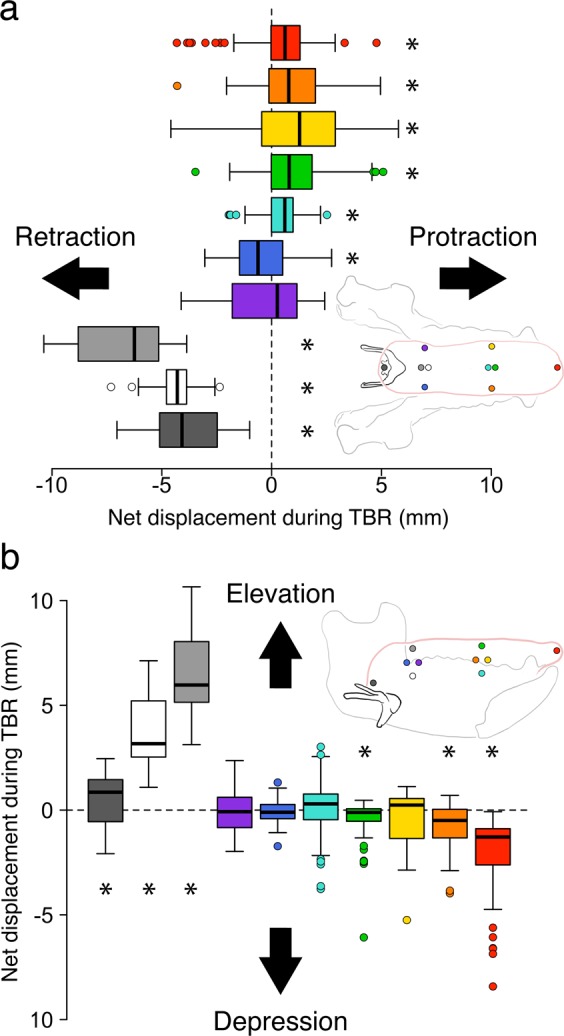
Tongue marker kinematics during tongue base retraction (TBR). Measurements were taken relative to the marker’s position at the start of TBR. Positive values indicate protraction or elevation; negative values indicate retraction or depression. All measures are in a cranial coordinate system. (**a)** Tongue marker anteroposterior displacement. (**b)** Tongue marker superoinferior displacement. Data are averaged across all monkeys. Boxes indicate the interquartile range, thick bars indicate median, error bars indicate data range, and circles are outliers. Asterisks indicate the mean is significantly different from zero using a one-sample Wilcoxon signed rank test. Arrows indicate marker trajectories. Colors correspond to the following markers: red, anterior; orange, right lateral; yellow, left lateral; green, middle surface; light blue, middle deep; dark blue, posterior right; purple, posterior left; gray, posterior surface; white, posterior deep; dark gray, vallecula. The pink outline of the tongue is based on diceCT (iodine-enhanced) data indicating the position of the markers relative to the mucosa. The same measurements taken in individual monkeys, as well as within a mandibular coordinate system, are included in Supplementary figures 1–5.

In primates, swallow jaw gape cycles consist of fast close, slow close, slow open, and fast open phases and are longer in duration than chewing cycles, primarily because of longer slow open and fast open phases^20,31^ (Fig. 2a). We defined tongue base retraction (TBR) onset as the start of posterior displacement of the posterior superficial tongue marker, and our data show that this began during slow open, in the intercuspal phase (IP), and was complete prior to or around the end of slow open^31^ (Fig. 2b). In all four individuals, TBR was characterized by retraction and elevation of *midline posterior* markers (posterior surface and deep, vallecula); if the *lateral* posterior tongue markers retracted and elevated at all, they did so significantly less than the posterior midline markers (p < 0.001) (Fig. 3a,b). The middle and anterior tongue markers protracted and depressed slightly as the mandible depressed by several degrees at the end of TBR. Tongue base retraction is a behavior of the midline posterior tongue, with little or no involvement of more anterior or lateral tongue (Figs. 3a, S1–5; Supplementary Videos 1 and 2).

Oral cavity volume decreased during the first part of TBR as the hyoid protracted and elevated (Fig. 2c,f), then increased late in TBR as jaw depression increased (Fig. 2c,a). Tongue base and posterior oral tongue volumes increased throughout TBR or increased and then plateaued late in TBR (Fig. 2c). Hyoid protraction and, to a lesser extent, elevation extended throughout TBR (Fig. 2f).

### Extrinsic tongue muscles do not shorten sufficiently to account for the magnitude of TBR

**Figure 4:**
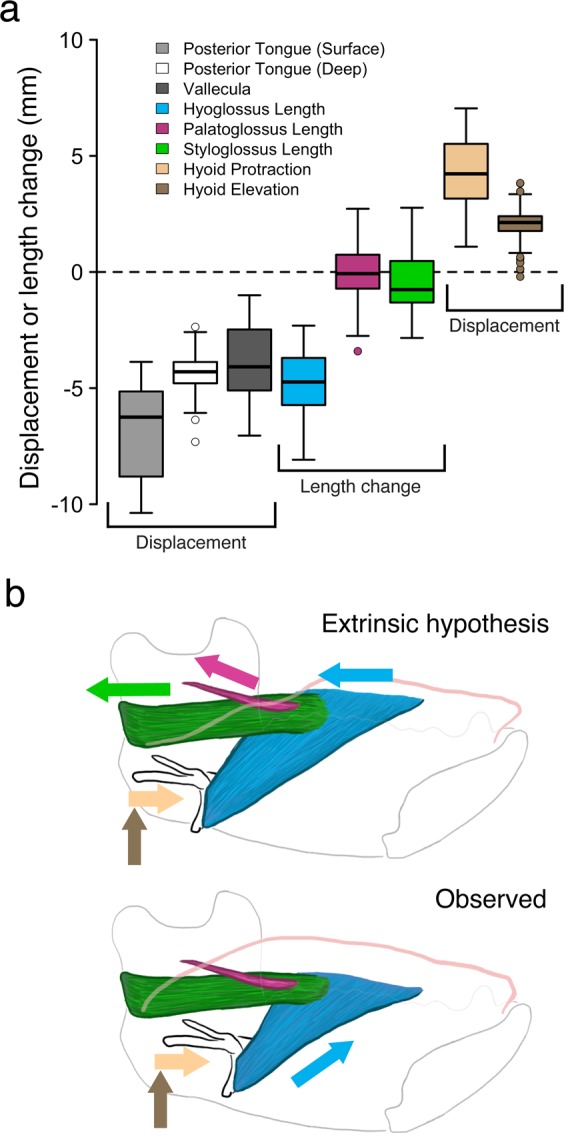
Hyolingual and muscle kinematics during tongue base retraction. All measures in a cranial coordinate system. (**a)** Box plot of mean displacements and extrinsic lingual muscle length changes. Positive values indicate hyoid protraction, hyoid elevation, or muscle lengthening; negative values indicate TBR or muscle shortening. Boxes indicate the interquartile range, thick bars indicate median, error bars indicate data range, circles are outliers. Arrows indicate the trajectory of hyoid movement and muscle shortening. All differences among tongue marker retraction and muscle length change were statistically significant after Bonferroni correction except for the differences between posterior deep and vallecular marker retraction and between posterior deep retraction and hyoglossus shortening in both mandibular and cranial coordinate systems. (**b)** Representative extrinsic muscle behavior and hyolingual posture at TBR onset and the displacements predicted by the extrinsic muscle hypothesis, contrasted with the observed extrinsic muscle length and hyolingual posture at the end of TBR.

The hyo-, palato-, and styloglossus muscles were predominantly horizontally oriented during TBR (135**°** <Θ_sagittal_ < 225**°**) (Table 2) and therefore were theoretically capable of producing the posteriorly-oriented forces necessary for TBR. However, if these muscles actually caused TBR they should have shortened a distance similar to TBR: they did not. Shortening distances of palatoglossus and styloglossus muscles were significantly less than retraction distance of the tongue base (p < 0.001) (Figs. 2b,d; 4a). The posterior surface, posterior deep, and vallecular markers retracted a median of 6.25 (± 1.88 s.d.), 4.30 (± 0.89), and 4.08 (± 1.57) mm, respectively. In contrast, the styloglossus muscle only shortened 0.76 (± 1.32) mm, significantly less than the amount of retraction observed in all three midline tongue base markers (p < 0.001). The palatoglossus muscle actually lengthened slightly (−0.07 ± 1.27 mm) (Fig. 2e). Hyoglossus muscle shortening (4.74 ± 1.30 mm) did approximate TBR in magnitude (Figs. 2e, 4a), but the right lateral tongue markers—implanted where the right hyoglossus muscle inserts into the tongue—actually protracted slightly, rather than retracting during TBR (0.93 ± 1.63 mm) (Fig. 3a). Therefore, rather than retracting the tongue, hyoglossus muscle shortening facilitated hyoid elevation (2.13 ± 0.71 mm) and protraction (4.23 ± 1.51 mm) (Fig. 4b) if active during swallowing. Moreover, if these extrinsic muscles were responsible for TBR then the lateral sides of the posterior tongue, where they insert, would have displaced backwards during TBR. But, as noted above (Fig. 3a), the lateral posterior tongue markers retracted minimally during TBR. In sum, our results falsify the hypothesis that extrinsic tongue muscle shortening causes tongue base retraction during swallowing.

**Table 2:** Muscle orientation (Θ_sagittal_) during tongue base retraction (cranial coordinate system).

Monkey	Hyoglossus		Palatoglossus		Styloglossus	
	Mean	Range	Mean	Range	Mean	Range
C	—	—	—	—	168.9°	151.02°–178.07°
H	227.6°	203.60°–252.99°	—	—	175.5°	151.20°–183.38°
J	211.9°	186.97°–222.94°	167.0°	131.81°–186.57°	167.7°	153.66°–183.63°
K	227.1°	212.27°–240.02°	137.8°	94.87°–174.57°	185.9°	169.86°–191.80°
Overall	223.2°	186.97°–252.99°	151.1°	94.87°–186.57°	174.4°	151.02°–191.80°

135**°** <Θ_sagittal_ < 225**°**; superior = 90°, posterior = 180°, inferior = 270°.

### Tongue base width changes minimally as its length and depth increase, with regional volume change

**Figure 5:**
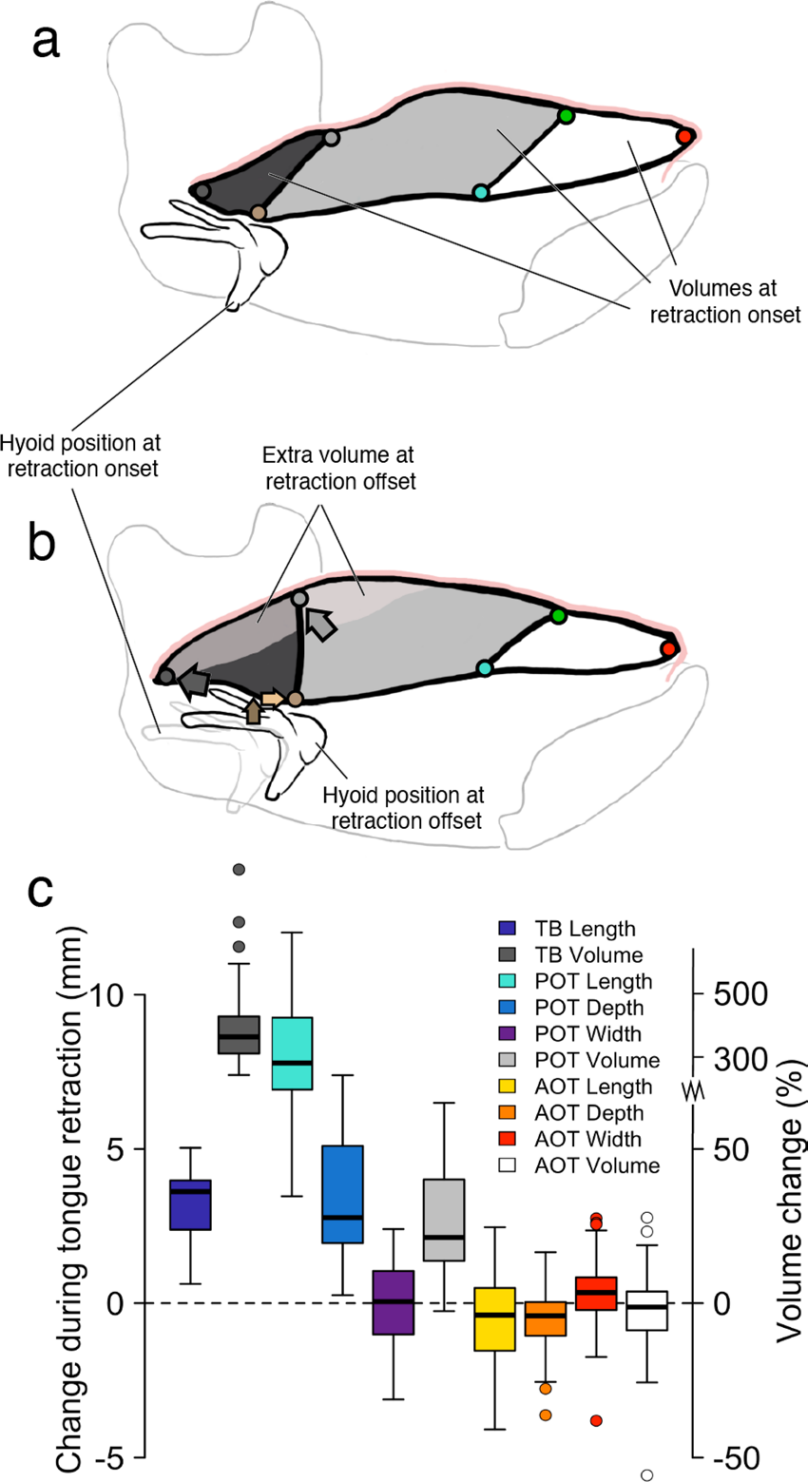
Change in tongue volumes during tongue base retraction (TBR). (**a**) Diagram illustrating midsagittal slices of anterior oral tongue (AOT), posterior oral tongue (POT) and tongue base (TB) volumes, and hyoid position prior to TBR. Tongue contours were traced from videoradiographic data of a macaque in lateral view at the beginning (A) and end (B) of TBR. Markers are: red, tongue tip; green, middle superficial; blue, middle deep; light gray, posterior superficial; brown, posterior deep; gray, vallecular. (**b**) Diagram illustrating changes in tongue shape, midline location of extra volume, and hyoid position at end of TBR. In A and B, marker colors correspond to those in Fig. 2, and the brown marker indicates the hyoid landmark used for TB and POT volume measurements. (**c**) Change in tongue linear dimensions and volumes during TBR.

If contraction of intrinsic tongue muscles causes TBR through a hydrostatic mechanism, then tongue base (TB) and posterior oral tongue (POT) volumes should remain constant and the tongue should decrease in width and/or height as the tongue base retracts. While anterior oral tongue (AOT) dimensions and volume remained relatively constant during TBR (width, 0.34 ± 1.02 mm; depth, −0.41 ± 0.95 mm; length, −0.39 ± 1.44 mm; volume, −0.01 ± 0.12 mL, −1.3% ± 11.5%), POT volume increased 0.85 mL ± 0.63 mL (21.3% ± 16.7%) and TB volume increased 0.65 mL ± 0.22 mL (361% ± 217%) during TBR (Figs. 2c, 5). The width of the common interface between TB and POT changed little (0.05 ± 1.37 mm), while TB length, POT length, and the depth of the TB-POT interface all increased (TB length, 3.61 mm ± 1.08 mm; POT length, 7.79 ± 1.71 mm; TM-POT interface depth, 2.77 ± 2.04 mm). At the end of TBR, the three monkeys with a marker in the vallecula (C, J, and K) demonstrated rapid increases in TB length as TB width and depth decreased slightly (Fig. 2d). However, in these animals TB *volume* continued to increase during late TBR (Fig. 2c), suggesting that increases in TB length—and, hence, TBR—were due to increased regional volume. Therefore, the muscular hydrostat model—in which tongue dimensions change reciprocally as regional volume is conserved—does not explain TBR.

### Tongue, hyoid, and muscle coordination suggest that suprahyoid muscles drive TBR

If TBR during swallowing is caused by a hydraulic mechanism involving the hyoid apparatus and tongue, then during TBR the hyoid should elevate and protract into the oral volume as suprahyoid muscles actively shorten, and oral volume should decrease while tongue base volume increases. As predicted, during swallow cycles, hyoid elevation and protraction began prior to, and continued throughout, TBR (Fig. 2f), whereas oral volume began decreasing prior to TBR, reached a minimum midway through TBR, then increased rapidly during the latter half of TBR in association with jaw depression (Fig. 2c). The low magnitude jaw depression characterizing the first half of TBR on its own would have increased oral volume, were it not for simultaneous hyoid protraction and elevation, which actually resulted in decreased oral volume (Fig. 2c,f). At the end of TBR the hyoid switched from elevation to depression, but continued to protract for a short time, before retracting during jaw depression (Fig. 2a,f).

**Figure 6:**
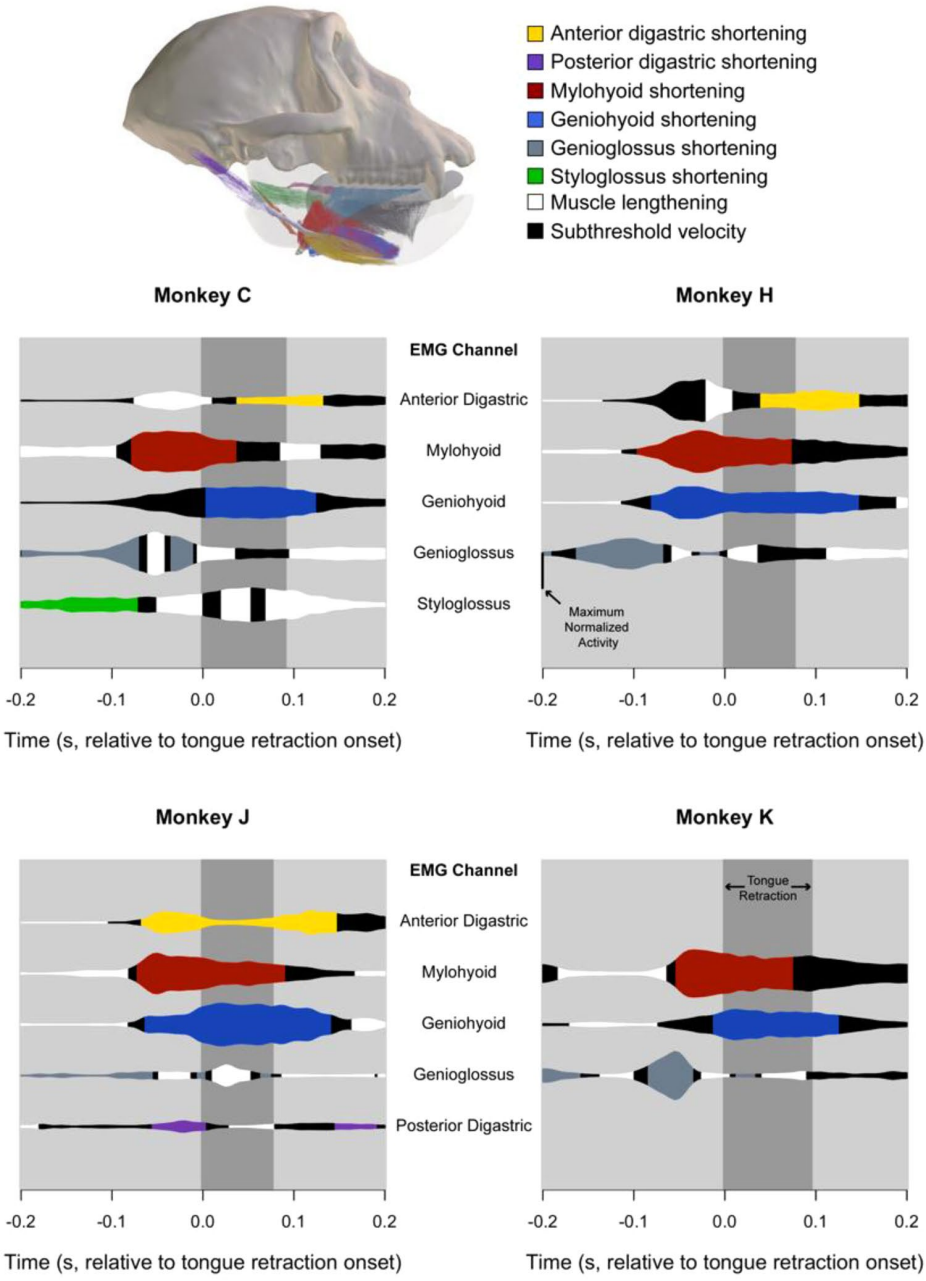
Hyolingual muscle activity and velocity during swallowing. EMG data have been rectified, integrated, and flipped over each’s axis to facilitate data visualization. The magnitude of each tracing indicates the magnitude of normalized muscle activity. Colors indicate that the respective muscle is shortening, white indicates muscle lengthening, and black indicates isometry. Data are not available for some animals because they either lacked markers (Monkey K digastrics) or EMG electrodes were confirmed implanted in only one animal (Monkey C styloglossus, Monkey J posterior digastric). Vertical dark gray bar indicates the duration of tongue base retraction. Data are aligned to the onset of tongue base retraction (Time = 0.0).

Data on muscle behavior—specificially activity and velocity—of genioglossus, geniohyoid, mylohyoid, digastrics, and styloglossus during swallowing are summarized in Fig. 6. Geniogossus exhibits low-level activity during TBR in all animals, and a distinct burst of activity in two animals. In three animals, maximum activity in genioglossus is observed before TBR as the genioglossus fibers shorten. These fibers subsequently lengthen and exhibit either low-level activity or a small burst of activity during TBR. Geniohyoid passively lengthens before TBR in all animals. Geniohyoid actively shortens during TBR and in three out of four animals TBR corresponds with peak geniohyoid activation. Posterior mylohyoid reaches peak activity and actively shortens and rotates in coronal planes before TBR. During TBR, posterior mylohyoid continues to shorten with less activity. In Monkeys C and H, the anterior digastric actively lengthens before TBR onset. As with mylohyoid, peak anterior digastric activity precedes TBR onset in three out of four animals. However, anterior and posterior digastric activity during TBR is minimal in one animal (Monkey J).

Muscle activity data were not available for hyoglossus or stylohyoid because electrode pairs were not confirmed to be within their muscle fascicles *post mortem* using diceCT. Hyoglossus shortens before and during TBR and rotates minimally to become slightly more superiorly oriented as the hyoid protracts. In contrast, stylohyoid shortens during hyoid elevation before TBR and lengthens slightly as the hyoid protracts during TBR. Throughout stylohyoid shortening and TBR, stylohyoid rotates in sagittal planes to become more posteriorly oriented.

A hydraulic mechanism of TBR was also suggested by a wave of tongue volume that moved posteriorly during TBR. Protraction and elevation of the hyoid were associated with rapid increases first in POT volume in the first half of TBR, then in TB volume during the second half of TBR (Fig. 2c). POT reached its maximum volume nearly synchronously with the minimum in oral volume, then declined slightly or plateaued as TB volume continued to increase through TBR (Fig. 2c). Increases in TB volume persisted for as long as the hyoid continued to protract and elevate; i.e., until shortly after the end of TBR (Fig. 2c,f). At the end of TBR, retraction of the vallecular tongue base marker (Fig. 2b) is explained by changes in TB volume (Fig. 2c) and shape (Fig. 2d). When POT volume was peaking and TB volume was just starting to increase, TB width and depth were at their maxima and TB length was at its minimum; i.e., the tongue base was short, high, and wide. As TB volume increased through the end of TBR, the TB changed shape—the length rapidly increased while the width and height remained largely unchanged or decreased slightly (Fig. 2d, Supplementary Video 3).

Our correlational analyses suggest that movements of superficial and deep parts of the tongue base are mechanically coupled, but the surface marker retracts and elevates further than the deep posterior marker. This suggests that TBR is achieved by backward rotation of the posterior midline tongue about an axis somewhere near the front of the hyoid. Moreover, the deep and superficial posterior markers and the vallecular marker all retract and elevate significantly further than the posterior lateral (left and right) markers during swallowing (Fig. 3). This requires that the TB experiences shear in coronal and transverse (horizontal) planes during TBR. The para-midline block of intrinsic lingual muscles oriented orthogonal and parallel to the long axis of the tongue^32,33^ may be moving as a block relative to the more lateral extrinsic tongue muscles—stylo-, hylo-, and palatoglossus. It is theoretically possible to modulate tongue stiffness in different planes using intrinsic muscle activity: importantly, the more posterior regions of the tongue, including the tongue base, probably have less variation in activity-dependent stiffness than other areas of the tongue because they have relatively less muscle and more connective and glandular tissue in both humans^34^ and macaques (Supplementary figure S6).

A mechanical linkage between hyoid and tongue base kinematics is supported by correlations between kinematic variables (Supplementary tables S1–4). From the start of TBR to the time of minimum oral volume, hyoid protraction in a cranial coordinate system was correlated with retraction of all tongue base markers (r values Hyoid X vs.: Post. Surf. X, −0.84; Vallec. X, −0.74; Post. Deep X, 0.−84; all p < 0.001). Across the entire TBR duration, hyoid elevation in a mandibular coordinate system is correlated with elevation of all tongue base markers (r values Hyoid Y vs.: Post. Sup. Y, 0.81; Vallec. Y, 0.90; Post. Deep Y, 0.82; all p < 0.001).

## Discussion

The data presented here contradict two prevailing hypotheses about the mechanism of tongue base retraction (TBR) during the oral phase of swallowing and support our novel hydraulic model. This model is based on our recognition of a mechanical linkage between TBR and hyoid protraction and elevation. In macaques and humans, the oral phase of swallowing involves a ‘squeeze back’ mechanism in which the contact point between the tongue and the palate moves posteriorly, squeezing the food bolus out of the oral cavity and into the oropharynx. As the bolus moves into the oropharynx, the hyoid protracts and elevates and the most posterior part of the tongue—the tongue base—retracts, presumably to squeeze and/or push the bolus into and across the oropharynx^35^. During late TBR, the hyoid continues to protract and, to a lesser degree, elevate as the TB decreases in depth and width and POT decreases slightly in volume. These observations suggest that the hyoid compresses the POT against the hard palate during late TBR and continues to cause TBR despite simultaneous increases in oral volume.

### Previous hypotheses

Our data refute the hypothesis that posterior extrinsic tongue muscles—styloglossus, palatoglossus, and/or hyoglossus muscles—produce TBR during swallowing. The macaque and human styloglossus muscles are active during swallowing^9,10^, but in our macaques the styloglossus muscle shortened far less than the tongue retracted during swallowing. Given the styloglossus muscle’s minimal shortening during TB retraction, we suggest that the styloglossus muscle may instead be active isometrically to prevent the hyoid’s protraction from dragging the tongue base anteriorly during early TBR. Moreover, the styloglossus muscle lies *lateral* to the greater and lesser horns of the hyoid in both humans and macaques^20,32^, which necessarily elevate and protract along with the hyoid during swallowing. Elevation and protraction of these structures during TBR would transmit anteriorly-oriented forces to the tongue *medial* to the styloglossus, making it implausible—if not impossible—for the styloglossus muscle to produce retraction of the midline tongue base.

Similar to the styloglossus muscle, hyoglossus muscle orientation includes posterior components, but its lateral insertion into the tongue does not actually move posteriorly during swallowing; rather, hyoglossus muscle contraction works with geniohyoid and mylohyoid to elevate and protract the hyoid^20^. This implies that there must be anteriorly directed components of force acting on the tongue to prevent hyoglossus muscle activity from pulling the tongue down and back. There are two likely sources of these forces: first, the genioglossus muscle, which exerts anteriorly directed forces on the tongue and is active during TBR (Supplementary figure S9), and second, forces generated by the greater and lesser horns of the hyoid on the lateral portions of the tongue base and posterior tongue as the hyoid protracts during tongue base retraction.

We also refute the hypothesis that intrinsic tongue muscles directly cause TBR. This hypothesis requires the tongue to remain isovolumetric during swallowing, in adherence to classic muscular hydrostat theory^14,15^. During TBR, anterior oral tongue (AOT) volume remains relatively constant, but the posterior oral tongue (POT) and then the base of the tongue (TB) increase in volume as the wave of tongue/palate contraction moves posteriorly^31,35^. Thus, another model must be used to explain the behavior of the macaque tongue during swallowing.

### The hydraulic hypothesis

Our data suggest that TBR during swallowing is produced through a novel hydraulic mechanism, changing shape and generating force by regionally displacing volume in ways not captured by a paradigm of static regional volumes, as assumed by the muscular hydrostat hypothesis^23,27^. In this mechanism, shortening of the suprahyoid muscles (including the hyoglossus muscle) protracts and elevates the hyoid and elevates the floor of the mouth (via the mylohyoid muscle) into the oral volume—the space bounded by palate, teeth, mandible and many suprahyoid muscles. Hyoid elevation by mylohyoid and hyoglossus muscle shortening ‘primes’ the tongue, and subsequent protraction of the hyoid by geniohyoid and anterior digastric shortening further compresses the soft tissues within the oral volume. Via regional displacement of tongue volume, this hyoid excursion extrudes the midline tongue base between the horns of the hyoid and into the oropharynx, along the path of least resistance. Essentially, we propose that the hyoid and mouth floor function as a piston-like apparatus for the POT and TB, which itself functions as a piston for the food bolus^1^.

Biological hydraulic systems can amplify force and displacement by varying the surface area of input and output forces, as is commonly done with automobile hydraulic brakes^27^. Given that the POT moves further (and up to 198% faster) than the hyoid, the proposed hyolingual hydraulic linkage apparently amplifies tongue displacement and velocity rather than force because the interface between the inferior tongue and the hyoid apparatus is larger than surface area of the tongue base.

### Lesion studies support a role for suprahyoid muscles in tongue base retraction

The hyolingual apparatus is innervated and mobilized by several nerves and muscles; however, few studies have examined the function of each of these nerves and muscles in isolation. The hypoglossal nerve carries motor axons to and proprioceptive dendrites from the intrinsic and extrinsic muscles of the tongue, as well as axons exiting at C1 traveling to geniohyoid and thyrohyoid^36^. Iatrogenic hypoglossal transection or palsy in humans and experimental transection in animals is not consistently associated with poor tongue base retraction, or even symptoms of dysphagia^37–41^. Some have linked hypoglossal nerve transection to decreased oropharyngeal pressures, but these studies involved transections of axons to geniohyoid and therefore do not assess swallowing function in lesions targeting the lingual muscles alone^42^. However, a palatal prosthesis, which decreases the volume of the oral cavity, recovered oropharyngeal pressure and possibly TBR, suggesting that suprahyoid muscles alone can produce TBR in the absence of lingual and geniohyoid muscle contractility. Other case reports have documented similar swallowing improvements in humans with bilateral hypoglossal injury^43,44^. However, it is uncertain whether these improvements result from the prosthesis’ compensation for atrophy-related volumetric losses or a novel compensatory motor pattern. A notable murine, mylohyoid-specific lesion study^45^ found transient reductions in pharyngeal bolus speed and swallow rate with persistently decreased bolus size, suggesting that injury to a single suprahyoid muscle can evoke pathological pharyngeal mechanics. However, this study did not control for the effects of surgery alone with a sham procedure, and it is unclear whether persistent reduction in bolus size is due to damage to neighboring muscles versus altered sensory perception, hyolingual excursion, or tongue base retraction. Further studies targeting individual muscles and nerves with sham controls are necessary to further evaluate individual muscles’ function.

### Does a hydraulic mechanism explain tongue base retraction during swallowing in humans?

Macaque hyolingual anatomy and kinematics resemble those of humans in a number of important ways, suggesting that the data collected here are relevant to mechanisms of swallowing in humans^31^. Previous workers have argued that the mechanism of TBR in humans involves synchronized shortening of styloglossus, hyoglossus, and transverse intrinsic lingual muscles^6–9^. Hyoglossus and styloglossus muscles in humans are active during swallowing^9,46^, though evidence of hyolingual muscle activity is not evidence of their shortening^17,19^. Our macaque data show that, if the styloglossus and palatoglossus muscles are active, they are essentially isometric during swallowing, likely due to the functional and anatomic reasons described above.

The hyoglossus and styloglossus muscles have lower activity during the Mendelsohn maneuver than after regular swallows;^9^ in this maneuver, the hyolaryngeal apparatus is maintained in an elevated position and the tongue base is retracted against the posterior pharyngeal wall after the swallow^47^. Decreased activity in these muscles during this maneuver suggests that, as in macaques, they may actually not be important drivers of TBR in humans. A hydraulic mechanism may be a better explanation of tongue retraction during the Mendelsohn maneuver—the hyoid’s semi-protracted and more elevated position during the maneuver^48^ may displace sufficient tongue bulk to keep the tongue retracted without additional activity from the styloglossus muscle.

In humans, as in our macaques, the tongue base greatly increases in depth and length during TBR^6^. Studies on humans have been limited to two dimensions, so increases in depth and length have been used to infer decreases in width “assuming that tongue muscle is incompressible (hence isochoric) and that out-of-plane shear strains are negligible”^6^. The results of our study suggest that, in macaques at least, TB and POT volumes *increase* during retraction (417% and 27.9%, respectively), and that these tongue regions shear in coronal and axial planes as the midline elevates and retracts while the lateral tongue remains relatively stationary. Human tongues must also shear in coronal and transverse planes because the midline of the tongue is capable of forming a depression between its lateral edges, presumably through genioglossus muscle activity^49,50^. Hence, assumptions regarding absence of intra-lingual shear during TBR may not be justified.

If suprahyoid muscles produce TBR through a hydraulic mechanism in humans, these muscles should be active during TBR. Several studies have demonstrated that palatal pressure generated by the tongue, suprahyoid muscle activity, and hyoid kinematics are temporally coordinated, with suprahyoid activity preceding pressure generated by the tongue and hyoid movement^51–55^. This limited evidence from the human literature supports the hypothesis that suprahyoid muscle activity and tongue kinematics and kinetics are temporally coordinated. These data are congruent with our suggestion that a hydraulic mechanism drives TBR in humans.

## Conclusions

Our data refute the hypothesis that tongue base retraction during swallowing is due to shortening of hyoglossus, palatoglossus, and styloglossus muscles. A role for hydrostatic deformation in early tongue base retraction is also refuted by our data showing that tongue base and posterior oral tongue volume increase as oral volume decreases, demonstrating that regional tongue deformation is more complicated than previously assumed by the muscular hydrostat hypothesis. These reciprocal changes in volume suggest that tongue retraction is primarily driven by a hydraulic linkage in which hyoid protraction and mouth floor and hyoid elevation essentially squeeze the midline posterior tongue base back into the oropharynx between the horns of the hyoid, driving the food bolus towards the esophagus. Available kinematic data from humans suggest that the TBR mechanism that we hypothesize for macaques also applies to TBR in humans. Based on this novel model of tongue base kinematics, emphasizing suprahyoid muscle training in patients with poor tongue base retraction may be more effective than treatments which target the tongue alone.

## Methods

### Ethical approval

The University of Chicago laboratory animal facilities are governed by federal regulations issued by the U.S. Department of Agriculture and the National Institutes of Health Office of Laboratory Animal Welfare. The University of Chicago has an approved assurance with the NIH, is registered with the USDA, and has Full Accreditation from the Association for Assessment and Accreditation of Laboratory Animal Care (AAALAC).

All experiments were performed in accordance with guidelines and regulations of the University of Chicago Institutional Animal Care and Use Committee (IACUC); full description of surgical procedures can be found in Orsbon *et al*.^20^. The animals were housed at an AAALAC-accredited animal facility attended to daily by veterinary and husbandry staff. For surgical procedures, anesthesia was induced with ketamine and maintained with inhaled isoflurane and were given two days of post-operative buprenorphine for analgesia. On non-training days, animals were fed monkey biscuits and given daily enrichment and *ad libitum* access to water.

### Data collection

Three-dimensional hyolingual kinematics were collected from two adult male and two adult female rhesus macaques (*Macaca mulatta*) at 200 Hz using biplanar videoradiography of implanted tantalum makers and the XROMM workflow: X-Ray Reconstruction of Moving Morphology^20,28^. In accordance with principles of reducing and reusing animals, all animals used in this study had previously been used in neurophysiological experiments involving neural array implantation in orofacial motor and sensory cortex (Monkey H, female, age 8, 7.46 kg), limb areas of sensorimotor cortex (Monkey K, female, age 12, 7.55 kg), parietal and premotor cortex (Monkey J, male, age 16, 8.48 kg), or prefrontal cortex (Monkey C, male, age 9, 8.83 kg). The animals were trained to feed while sitting in an X-ray lucent, acrylic primate chair. On training days, the animals were sometimes food- and water-delayed until after data collection. Radiation doses were approximately 1,500 μSv for every 50 seconds of data collection based on handheld dosimetry, which is slightly less than the radiation dose of a head CT scan (2,000 μSv). Many swallows could not be analyzed due to obscured markers or movement outside of the capture volume. A total of 99 swallows of red grapes was included in the final analysis. Analysis was limited to a single food type (red table grapes) that was very palatable to all animals to minimize variation in response to variable food material properties.

Markers were implanted in the cranium, mandible, and hyoid following the surgical procedures described by Orsbon *et al*.^20^. Of note, biologically inert markers implanted into the oropharyngeal soft tissues of dogs maintain their position over time, likely due to acellular, fibrous encapsulation^56^. Supplementary figure S7 shows the constellation of markers implanted in the tongues of all four animals. Seven markers were implanted under the dorsal tongue surface. Four were implanted under the lateral dorsal tongue surface, bilaterally at the junction of the palatoglossal arch with the tongue and at the lateral border of the tongue, and halfway between the posterior-most circumvallate papillae and the tongue tip. Three were implanted in the midline dorsal tongue surface: one in the tongue tip, one between the posterior-most circumvallate papillae, and one halfway between these two. Two additional midline markers were more deeply implanted, about 10–20 mm deep to the dorsal surface: one at the posterior-most circumvallate papillae, and another at halfway to the tip of the tongue. The overall constellation of markers approximates a pyramidal shape when the tongue is at rest. A marker was also implanted in the tongue base (i.e., the lingual side of the valleculae) in each animal, however in Monkey H this marker was extruded during healing. Monkey K’s posterior deep tongue marker was found to be immediately adjacent to the basihyoid so that its kinematics closely mirrored those of the hyoid.

Extrinsic lingual muscle length and orientation were measured relative to the plane of the palate (anterior = 0°)^20^. Muscle length was defined as the Euclidean distance between two markers within a muscle, a marker and a reconstructed point, or two reconstructed points. A muscle was defined as having a predominantly posterior orientation when its sagittal angle (Θ_sagittal_) relative to the maxillary molars was greater than 135 and less than 225 degrees. Intrinsic muscle kinematics were inferred from changes in tongue dimensions measured as the Euclidean distance between lingual markers projected onto the anatomical coordinate system: posterior tongue height was the Y-axis (vertical) distance between the posterior surface marker and the dorsum of the hyoid; posterior tongue width was the Z-axis (mediolateral) distance between the two lateral posterior markers; posterior tongue length was the X-axis (anteroposterior) distance between the posterior and middle surface markers.

### Regional tongue volume

Although increases in all three dimensions of the posterior tongue would necessarily support the conclusion that the tongue regionally changes in volume, it is possible for volume to increase even if one or two dimensions decrease in size. Therefore, regional tongue volume was also modeled. Simple convex models have the advantage of computational simplicity, but the tongue can form distinctly concave shapes^49,50^. For each frame, a 3D volume was created by generating a polygonal mesh using a geometry processing algorithm developed by TG. The algorithm models the tongue as a concave shape consisting of three geometric primitives delimited by two coronal surfaces generated from instantaneous tongue posture (Supplementary figure S8). The two surfaces were constructed from two closed spline curves each interpolating a set of four implanted markers, respectively. The *middle coronal surface* at the border between the anterior and posterior oral tongue was defined by the middle superficial, middle right, middle left, middle deep markers. The *posterior coronal surface* at the border between the posterior oral tongue and the tongue base was defined by the posterior superficial, posterior right, and posterior left markers as well as a digitally reconstructed marker on the dorsal surface of the hyoid (see Brainerd *et al*.^28^, Menegaz *et al*.^30^, and Orsbon *et al*.^20^ for detailed methods of digital marker reconstruction). The posterior deep marker of the tongue was not used because of significant disparities in posterior deep marker position among animals compared to the more regularly spaced superficial markers, and because the hyoid adequately delimits the inferior border of the tongue.

Splines were fit using the splinefun function of the R base package with the method set to “natural”. For the sake of stability, each coronal surface was determined by initially fitting the corresponding set of four implanted markers using a natural spline that wraps around the ordered set with three full loops of 300 points each, starting and ending at the middle deep or dorsal hyoid marker for the middle and posterior coronal splines, respectively. Then, the segments of the spline outside of the second complete loop were discarded. A minimal surface^57^ was then constructed for each coronal surface by solving a minimal surface equation with the x-, y-, and z- coordinates of the coronals as boundary conditions. For this purpose, first each coronal surface was projected onto a two-dimensional plane spanned by the first two principal directions, then a triangular mesh was constructed for the planar domain enclosed by the projected image, and finally a system of three Poisson equations defined on the planar triangular mesh was solved to obtain the x-, y-, and z-coordinate functions for the minimal surface. This methodology closely follows the surface editing algorithm based on cotangent Laplacians on irregular triangular meshes^58^. The minimal surfaces enclosed by the middle and posterior coronal splines were the *middle and posterior coronal surfaces*, respectively. Note that although each minimal surface was constructed by solving planar Poisson equations, the resulting surfaces were not flat unless the boundary curve actually resided in a plane, since the boundary conditions are determined by the actual spatial configuration of the coronal splines.

The two coronal surfaces divided the tongue into three adjacent portions. For each, a triangular mesh was constructed with the shape of a geometric primitive and its 3D mesh volume was calculated as the sum of all the signed volumes^59^ of the tetrahedrons spanned by the triangular faces with respect to an arbitrarily fixed origin. The total sum of the signed volumes did not affect the final result, but the centroid of the corresponding geometric primitive was picked for definiteness. The three geometric primitives were:

1. *Anterior oral tongue (AOT)*: A cone with the middle coronal surface as its base and the anterior tongue marker as apex;
2. *Posterior oral tongue (POT)*: A deformed cylinder with the middle and posterior coronal areas as two bases;
3. *Tongue base (TB)*: A cone with the posterior coronal area as base and the vallecula marker as apex.

To generate surface meshes for the three geometric primitives in a meaningful way, the middle and posterior coronal splines were resampled in a consistent manner. First, a sequence of new sample points was generated on the middle coronal spline that evenly divided the spline into segments of equal arc-length. Then, for each new sample, a corresponding point on the posterior coronal spline was generated in such a way that the new samples on the posterior coronal spline also divided the spline into segments of equal arc-length. Finally, the middle deep marker was matched with the reconstructed dorsal hyoid marker. Equivalently, the resampling procedure can also be viewed as first matching the middle deep marker with the reconstructed dorsal hyoid marker and then generating an equal number of evenly distributed new samples on each spline; the new samples on the two coronal splines are in canonical one-to-one correspondence determined by clockwise or counter-clockwise ordering, with respect to the initial matching between the middle deep marker and the reconstructed dorsal hyoid marker. The AOT and TB cone meshes were generated by connecting the apex with each new sample on the corresponding spline with line segments, while the POT cylinder was constructed by simply adding line segments connecting pairs of matched new samples. All three meshes were then refined by subdividing the line segments and filling the gaps between neighboring line segments with small triangle faces.

This regional volume measured is necessarily smaller than the entire volume of the tongue for two reasons. First, the markers are placed below the surface of the tongue. Second, to facilitate data processing speed, only 10 markers were placed as these would capture of the bulk of tongue movements while sacrificing measuring the tongue as a whole. Figure S8 compares the regional volume models with that of the entire tongue.

Oral cavity volume was measured using a 3D alpha shape (Supplementary figure S8). The oral cavity was defined as the space bounded superiorly by the mucosa of the hard palate; anteriorly and laterally by the lingual surfaces of the teeth, mandibular symphysis, and mandibular corpus as far inferiorly as the mandibular attachment for the mylohyoid; inferiorly by a reconstructed mylohyoid raphe running from the inferior pole of the hyoid to the mandibular symphysis immediately inferior to the origin of geniohyoid; and posteroinferiorly by the anterior surface of the hyoid. These boundaries were assumed to be either rigid (hard palate, teeth, mandible, and hyoid) or linear (mylohyoid raphe). Using Autodesk MeshMixer 2018 (Autodesk, San Rafael CA), OBJs of each of these rigid surfaces was created from the hyoid and mandible bone models as well as a model of the segmented cranial teeth and hard palate mucosa. The kinematics of 4684–8308 total bony landmarks were then reconstructed using the XROMM workflow and served as the input values for the alpha shape function in the alphashape3D package in R (v1.3^60^). Volumetric measurements were taken from the resulting alpha shape for each frame.

### Electromyography data acquisition and processing

Insulated, medical-grade stranded stainless steel fine-wire electrodes (Cooner Wire, Chatsworth CA) were implanted for EMG recordings. A 27-pin connector (Omnetics Connector Corporation, Minneapolis MN) was housed in a custom-built, percutaneous, titanium housing that was rigidly fixed to the cranium using bone screws. The EMG connector allowed for recording from 13 muscles using four differential amplifiers (Model 1700, AM Systems, Sequim WA), and the 27th wire remained implanted under the skin as a ground wire. The wires were tunneled subcutaneously to the submandibular area and 1–2 mm of insulation was stripped. Electrodes were implanted into the muscle using an 18- or 20-gauge needle and were stimulated through the connector intraoperatively to confirm their location (single stimulation, train rate = 1 Hz, train duration = 300 ms, stim rate = 250 pulses per second, delay = 0.01 ms, duration = 0.2 ms, voltage = 5–10 V, Grass S48 Stimulator, AstroNova, Inc., West Warwick RI). Signals were amplified with differential amplifiers (AM Systems Model 1700) and synchronized with videoradiography data using a video processing unit (XCitex ProCapture VPU).

Electromyography (EMG) data from all animals were processed using a 30 Hz high-pass Butterworth filter that filtered forward and back to eliminate phase shifts using the *butter* and *filtfilt* functions of the signal R package^61^. We used different low-pass cutoffs among the different animals because EMG data were collected at different sampling rates (2000–10000 Hz). Low-pass cutoffs ranged from 1000 to 3000 Hz. Filtered data were full-wave rectified and integrated using a root-means squared algorithm integrated over 5 ms intervals, which also functioned to down sample the data to match the cineradiography frame rate of 200 Hz.

Each channel’s noise threshold was determined using a method similar to that proposed by Thexton^62^. In brief, Thexton’s method uses a runs test to determine the noise threshold, which involves comparing ordered and randomized EMG data. At low thresholds, noise leads to an increased number of threshold crossings (i.e., separate runs) in ordered and randomized data as time progresses. However, at higher thresholds, the number of runs in ordered EMG data will be limited to the sum of the number of separate activity and noise bouts but in randomized EMG data will decrease at a slower rate. The selected threshold optimizes the difference in run number between ordered and randomized EMG data. Our method differed in that we used an average threshold of 30 runs tests for each channel, and a threshold from a temporal subset of each trial in some channels. For each channel, a trial in which the animal exhibited clear periods of activity and noise was chosen for the runs test. In some channels, the entire trial was used. However, when the total duration of activity exceeded that of noise, the algorithm produced a threshold that cut off salient bursts of activity. In these circumstances, the input data for the runs test was narrowed to a smaller window in which the total duration of activity was roughly equal to that of noise. After determining the channel’s threshold in the trial used for the runs test, this threshold was applied to the other trials in the dataset. Every channel was visually inspected for a goodness of fit before applying the threshold to all trials.

To measure the amount of concentric, isometric, and eccentric activity in each cycle and tongue base retraction, muscle velocity was averaged across all cycles. For each of these frames with supra-suprathreshold muscle activity, muscle velocity was measured as the first derivative of muscle length waveforms—negative velocities indicated shortening, while positive velocities indicated lengthening. Isometry was indicated by velocities that were within two standard deviations of precision study velocity of zero as described by Menegaz *et al*.^30^.

### Temporal alignment

Changes in extrinsic muscle length, tongue dimensions, and regional tongue volume were measured as the difference in their values between the onset of tongue base retraction (TBR), the time at which the posterior superficial marker was at its most anterior point before beginning to move posteriorly, and TBR offset, the time at which the vallecular marker was at its most posterior point after tongue base retraction onset. Intercuspal phase (IP) was defined as the period when mandibular pitch velocity was less than two standard deviations from each animal’s precision study zero velocity.

### Statistical analyses

All statistical comparisons were conducted in the R base package using either a one-sample or paired Wilcoxon signed rank test using the wilcox.test base function, given that the data were not normally distributed. A p-value of less than 0.05 was considered statistically significant. Pearson correlation matrices were generated from hyolingual kinematic variables using the cor.table function of the picante package in R^63^. Threshold p-values were adjusted in multiple comparisons using a Bonferroni correction.

### Limitations

The time-intensive nature of data processing limited data collection to four animals (two males, two females) and one food type (red grapes) which macaques are motivated to eat. Whether the same conclusions apply to swallowing of other foods remains to be determined. Some measurements of muscle length and tongue kinematics were not possible in all animals, although most variables were measured in at least two animals. In Monkey C, the palatal palatoglossus muscle marker and lingual hyoglossus muscle markers were not implanted close enough to the muscle to justify using these markers to measure muscle lengths. In Monkey H, palatal palatoglossus muscle markers were not implanted due to concerns that these markers would occlude tongue markers in future neurophysiological experiments; and Monkey H’s vallecular marker extruded during healing. In Monkey K, the posterior deep marker was implanted more deeply than the other markers and was adjacent to the hyoid. Consequently, its kinematics were very different from the same marker in other animals (Supplementary figures 2–5, but were similar to a similarly placed marker reported by Nakamura *et al*.^31^.

It is possible that prior neurophysiological studies may have affected swallowing biomechanics in these animals. However, although Monkey H had electrodes in orofacial primary sensorimotor cortex, this monkey’s kinematics were broadly similar to monkeys with prior electrodes in regions remote from the orofacial primary sensorimotor cortex, specifically the limb regions of sensorimotor cortex (Monkey K), parietal and premotor cortex (Monkey J), or prefrontal cortex (Monkey C) given the focus on upper limb motor control in those studies.

Our model based volumetric estimates would benefit from further refinement. In particular, increases in TB volume may be overestimated, considering that the dorsal hyoid was used as a landmark to define the boundary between the TB and POT. Therefore, parts of TB volume are a product of not only TBR but also hyoid protraction. However, TB length increases from mid-TBR until shortly after TBR offset and was measured as the anteroposterior distance between the posterolateral and vallecular markers. Therefore, any artifacts introduced by using the hyoid as a boundary between the TB and POT likely only exaggerate the trends identified here, rather than reversing them.

## Supplementary information

Supplemenatry information.

Supplemenatry information.

Supplemenatry information.

## Data Availability

Data are available on the University of Chicago’s XROMM data management portal by request.
